# Delivery of biannual ultrasound surveillance for individuals with cirrhosis and cured hepatitis C in the UK

**DOI:** 10.1111/liv.15528

**Published:** 2023-02-20

**Authors:** Victoria Hamill, Will Gelson, Douglas MacDonald, Paul Richardson, Stephen D. Ryder, Mark Aldersley, Stuart McPherson, Sumita Verma, Rohini Sharma, Sharon Hutchinson, Jennifer Benselin, Eleanor Barnes, Indra Neil Guha, William L. Irving, Hamish Innes

**Affiliations:** ^1^ School of Health and Life Sciences Glasgow Caledonian University Glasgow UK; ^2^ Public Health Scotland Glasgow UK; ^3^ Cambridge Liver Unit Cambridge University Hospitals NHS Foundation Trust Cambridge UK; ^4^ Gastroenteology and Hepatology Royal Free London NHS Foundation Trust London UK; ^5^ Royal Liverpool and Broadgreen University Hospitals NHS Trust Liverpool UK; ^6^ NIHR Nottingham Biomedical Research Centre Nottingham University Hospitals NHS Trust and the University of Nottingham UK; ^7^ Leeds Liver Unit St James's University Hospital Leeds UK; ^8^ Newcastle Liver Unit Freeman Hospital Newcastle upon Tyne UK; ^9^ Department of Clinical and Experimental Medicine Brighton and Sussex Medical School Brighton UK; ^10^ Department of Gastroenterology and Hepatology University Hospital Sussex NHS Foundation Trust Brighton UK; ^11^ Imperial College London London UK; ^12^ Nuffield Department of Medicine and the Oxford NIHR Biomedical Research Centre University of Oxford Oxford UK; ^13^ Nottingham Digestive Diseases Centre, School of Medicine University of Nottingham Nottingham UK; ^14^ Division of Epidemiology and Public Health University of Nottingham Nottingham UK

**Keywords:** adherence, imaging, liver cancer, screening, ultrasonography

## Abstract

**Background:**

Previous studies show the uptake of biannual ultrasound (US) surveillance in patients with cirrhosis is suboptimal. Here, our goal was to understand in broader terms how surveillance is being delivered to cirrhosis patients with cured hepatitis C in the UK.

**Methods:**

Hepatitis C cirrhosis patients achieving a sustained viral response (SVR) to antiviral therapies were identified from the national Hepatitis‐C‐Research‐UK resource. Data on (i) liver/abdominal US examinations, (ii) HCC diagnoses, and (iii) HCC curative treatment were obtained through record‐linkage to national health registries. The rate of US uptake was calculated by dividing the number of US episodes by follow‐up time.

**Results:**

A total of 1908 cirrhosis patients from 31 liver centres were followed for 3.8 (IQR: 3.4–4.9) years. Overall, 10 396 liver/abdominal USs were identified. The proportion with biannual US was 19% in the first 3 years after SVR and 9% for all follow‐up years. Higher uptake of biannual US was associated with attending a liver transplant centre; older age and cirrhosis decompensation. Funnel plot analysis indicated significant inter‐centre variability in biannual US uptake, with 6/29 centres outside control limits. Incident HCC occurred in 133 patients, of which 49/133 (37%) were treated with curative intent. The number of US episodes in the two years prior to HCC diagnosis was significantly associated with higher odds of curative‐intent treatment (aOR: 1.53; 95% CI: 1.12–2,09; *p* = .007).

**Conclusions:**

This study provides novel data on the cascade of care for HCC in the UK. Our findings suggest biannual US is poorly targeted, inefficient and is not being delivered equitably to all patients.

**Key points**

- HCC surveillance is inefficient (i.e., >10 000 scans performed in this cohort to treat only 49 HCC patients with curative‐intent).
- There is inequity in how surveillance is implemented between liver centres.
- Patients being prioritised for surveillance are not ideal candidates for curative‐intent treatment.
- The number of ultrasound episodes received in the two years prior to HCC diagnosis is associated with greater odds of curative‐intent treatment.

## 1 INTRODUCTION

The number of hepatitis C virus (HCV)‐infected individuals achieving a sustained virological response (SVR) has increased rapidly since the introduction of direct‐acting antivirals (DAAs).^1^ Achieving SVR is associated with diverse benefits^2^, ^3^; however, it does not completely eliminate the risk of hepatocellular carcinoma (HCC). Indeed, a recent meta‐analysis reported the incidence rate is 2.1 HCCs per 100 person years of follow‐up among cirrhosis patients following SVR.^4^

HCC is a leading cause of cancer mortality worldwide, killing ~0.8 million people every year.^5^ However, patients can have a favourable prognosis if treated with curative intent (i.e., via liver transplantation, surgical resection or ablation). Unfortunately, suitability for these treatment hinges on early HCC detection. Thus, because the majority of HCCs are not detected until an advanced stage, only about 4 patients in 10 go onto be treated with curative intent in the UK and other countries.^1^, ^6^

Clinical guidelines recommend individuals with cirrhosis should receive biannual ultrasound (US) of the liver/abdomen to maximise early HCC detection.^7^, ^8^, ^9^ However, a recent systematic review reported that only 9.8% of cirrhosis patients receive biannual US surveillance, based mainly on data from North America.^10^ At present, there is little detailed information regarding how surveillance is implemented in a real‐world cohort, particularly in a European setting. On this note, population‐based studies comprising a representative set of screening providers are crucial to build an accurate picture of surveillance practice as a whole. In the present study therefore, we used record‐linkage methods to integrate data on US exams, HCC incidence and curative HCC treatment, within a large multicentre cohort. Our goal was to study in detail how biannual US is implemented and patterned for HCV cirrhosis patients following SVR achievement.

## 2 METHODS

### 2.1 The HCVRUK resource

This study is underpinned by data from the Hepatitis C Research UK (HCVRUK) resource, a database of almost 12 000 patients with chronic HCV.^11^ HCVRUK participants were recruited from 2012 to 2015 from more than 50 UK liver centres. Participants have been characterised in terms of a broad range of clinical, epidemiological, virological and treatment‐related factors, ascertained through clinical notes or through direct self‐report at study enrolment.

More recently, a subset of the cohort – all participants with a cirrhosis diagnosis – have been linked to nationwide registries held by NHS Digital (application number: NIC‐72626) This includes hospital episodes statistics (HES) data (e.g., admitted patient care database^12^; diagnostic imaging dataset (DID)^13^ and outpatient hospital admissions), mortality registrations and the NCRAS cancer registry.^14^ Informed consent was obtained from all participants.

### 2.2 Study population: eligibility criteria

All HCVRUK participants diagnosed with liver cirrhosis who subsequently went on to achieve SVR through antiviral therapy were eligible for inclusion in the current analysis. Liver cirrhosis was defined as compensated or decompensated cirrhosis diagnosed during routine clinical investigation. In practice, diagnoses of cirrhosis were typically made following: liver biopsy; transient elastography; abdominal US; clinical examination; symptoms consistent with a decompensation episode; and routine liver function tests, according to clinical guidelines.

### 2.3 Study population: exclusion criteria

Eligible patients were excluded for (i) pre‐SVR liver transplant, (ii) a pre‐SVR HCC diagnosis, (iii) missing identifiers for record linkage, and (iv) <12 months of follow‐up after SVR achievement.

### 2.4 Ultrasound data

Data on abdominal/liver US examinations performed after SVR achievement were ascertained through NHS digital data. Two specific registries were used to capture imaging events: First, the diagnostic imaging dataset (DID), which provides patient‐level information on radiology scans performed in NHS England for diagnostic purposes. DID data are derived from local radiology information systems, which are collated by clinical commissioning groups, and submitted monthly to NHS Digital.^13^ Modality and body site of radiology scans are indicated through SNOMED‐CT codes. In this study, we selected US procedures performed specifically on either the liver or the abdomen using the SNOMED‐CT codes listed in Table S1. Data from the DID were then supplemented with the HES outpatient data, which provides information on outpatient hospital visits attended in NHS England. For this database, abdominal/liver US events were identified using the *U082* OPCS4 code (Table S1). At the time of analysis, both the out‐patient dataset and the DID were complete until 31 March 2020. In a sensitivity analysis, we also included magnetic resonance imaging (MRI) and computed‐tomography (CT) scans of the liver and/or abdomen to see what impact this had on surveillance uptake.

Please note, medical indications for imaging procedures are not recorded in these data registries and so were unavailable in this study.

### 2.5 Study follow‐up period

For each patient, the follow‐up period began at the date of SVR, defined as 12 weeks after treatment completion (i.e., SVR12). However, if a patient achieved SVR before enrolment into HCVRUK study, then we commenced follow‐up time at the date of study enrolment to avoid immortal time bias.

Follow‐up ended at the earliest of (i) date of liver transplant (if at all); (ii) diagnosis of HCC (if at all); (iii) date of death (if at all); or (iv) the study completion date of 31st December 2019.

Information on liver transplantation, HCC diagnosis and date of death was ascertained through NHS digital registries. The specific code sets used to identify these events are indicated in Table S1.

### 2.6 Ultrasound event vs ultrasound episode

We distinguished between US *events* (i.e., single liver/abdominal US) and US *episodes* (i.e., a cluster of US events relating to a single US episode). Thus, where patients had multiple US events within a 90‐day period, these were collapsed into a single US episode, with the earliest scan date retained. This step was to avoid overestimating uptake in patients with a single US episode entailing immediate follow‐up scans. Sensitivity analyses were performed exploring the impact of using a longer (150 days) and shorter time window (30 days) than 90 days.

### 2.7 Primary outcome event

The primary outcome event was biannual US, defined as a rate of ≥2.0 US per year. The rate of US uptake was calculated by dividing the total number of US episodes by the total follow‐up time per patient. Patients without biannual US were separated into three groups:

- Annual US: rate of ≥1.0 but <2.0 US episodes per year
- Infrequent US: rate of <1.0 but >0 US episodes per year.
- No US: zero US episodes during follow‐up.

In sensitivity analyses, more lenient definitions of biannual US were considered: (a) > 1.85 USs per year (1 scan per 6.5 months); and (b) 1.71 US per year (1 US per 7 months).

### 2.8 Study covariates

Study covariates were ascertained from two sources. First, information recorded directly on the HCVRUK clinical databases; second, hospital admission records occurring prior to SVR achievement (based on the hospital episodes statistics Admitted Patient Care dataset^12^).

Study covariates ascertained from the HCVRUK clinical database were age; gender; ethnicity (Caucasian and non‐Caucasian/unknown); decompensated cirrhosis; risk of HCC; and attending a liver transplant centre at HCVRUK recruitment. Baseline decompensation was defined as a decompensation event (ascites, bleeding varices or encephalopathy) before SVR. All dates of decompensation episodes were ascertained from the HCVRUK database. HCC risk at baseline was estimated using the aMAP score,^15^ which is calculated from information on age, gender, albumin, bilirubin and platelet count. The aMAP risk score was chosen because a previous validation analysis indicated that it had better discrimination and calibration in this patient group than rival HCC risk scores.^16^ When calculating aMAP, albumin, bilirubin and platelet count values were determined from test results performed up to a year before treatment initiation. If more than one test was performed during this window, then the mean value was used. These methods were used for consistency with previous studies and because antiviral therapy can cause temporary changes in laboratory tests that may not reflect long‐term risk profile.

Two covariates were derived from a patient's hospital admission history. These were (a) previous substance use‐related hospital admission; and (b) previous alcohol use‐related hospital admission. Three levels were considered for both variables: (1) no previous admission; (2) non‐recent admission (defined as more than 3 years prior to SVR); and (3) recent admission (defined as less than three years prior to SVR). The ICD codes used to identify these events are provided in Table S1.

### 2.9 Factors associated with biannual US

Logistic regression was used to identify factors associated with receiving biannual US. Candidate predictors assessed in univariable and multivariable models included age (per ten‐year increase); sex (male vs. female); ethnicity (Caucasian vs. non‐Caucasian/unknown); previous alcohol‐related hospital admission(s) (recent, and not recent vs. no); previous drug‐related hospital admission (recent, and not recent vs. no); decompensated cirrhosis (yes vs. no); and attendance at a liver transplant centre (yes vs. no). Duration of follow‐up can also affect biannual screening uptake, as it is easier to be adherent over a two‐year duration vs. a four‐year duration, for example. Thus, duration of follow‐up was also included as a covariate, which functioned as a type of offset in the model.

### 2.10 Variability in biannual US across individual clinics

Funnel plots were constructed to assess variation in biannual US uptake between individual centres. A funnel plot comprises a series of data points, one for each liver centre represented in our cohort.^17^ The vertical position of each data point reflects the crude proportion of patients who received biannual US at the centre in question; the horizontal position reflects the clinic's sample size. In the absence of inter‐clinic heterogeneity, all data points should move towards convergence as sample size increases. The binomial distribution was used to generate 95% and 99% control limits; centres outside these limits can be considered to exhibit atypical uptake that is unlikely to reflect sampling error.

### 2.11 HCC incidence rate

HCC cases were defined as a cancer, mortality or an inpatient hospital admission for HCC (ICD10: C22.0). The incidence rate of HCC was calculated by dividing the number of incident events by the study follow‐up period.

### 2.12 Association between US uptake and curative HCC treatment

For individuals with HCC, we calculated the number of US episodes performed in the two years prior to their HCC diagnosis. We then determined the association between the number of US episodes in that period and the odds of being treated for HCC with curative intent.

Curative‐intent treatment for HCC was defined as ablation, resection or liver transplantation, according to clinical guidelines.^8^ OPCS4 codes in the HES admitted patient care dataset were used to identify these instances of curative‐intent treatment (Table S1).

A logistic regression model was fitted to identify factors associated with curative treatment. In addition to the number of US episodes. Other covariates included in this model were age at SVR, gender, and decompensated cirrhosis at SVR.

### 2.13 Validation

Internal and external validation approaches was performed to assess if NHS digital data can reliably measure US uptake in cirrhosis patients.

Two types of internal validation were carried out. First, the average time interval between consecutive US scans was calculated to assess consistency with the screening interval recommended in clinical guidelines. Second, we assessed the timing of imaging examinations in patients who developed HCC. Our expectation was that there would be a spike in imaging procedures performed on/around the date of HCC diagnosis.

For external validation, we collected information on liver/abdominal US directly from liver centres for a subset of patients. These data were used to assess agreement between US uptake inferred from NHS digital data versus US inferred directly from liver centres. For more information, see Appendix A.

## 3 RESULTS

### 3.1 Derivation of the study population

**FIGURE 1:**
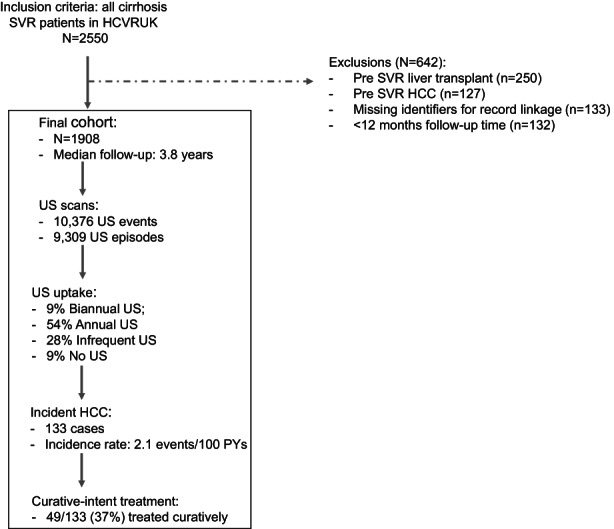
HCC care cascade.

A total of 2550 patients met our study inclusion criteria. We then excluded individuals if they had a pre‐SVR liver transplant (*n* = 250, 11%); had a pre‐SVR diagnosis of HCC (*n* = 127; 6%); were missing identifiers for record linkage (*n* = 133; 6%); or had less than 12 months follow‐up (*n* = 132, 6%). Thus, our final study population comprised the remaining 1908 patients (Figure 1).

### 3.2 Characteristics of study population

The study population were recruited from 31 liver centres covering all major geographical regions in England (Figure S1). Individuals were mostly middle‐aged (mean age: 55.0), male (74%) and Caucasian (81%). One‐fifth had a previous episode of decompensated cirrhosis (19.9%). The proportion with a recent hospital admission for substance use and alcohol use was 24% and 12% respectively. The median aMAP score at SVR achievement was 61.3, equivalent to a predicted 3‐year HCC probability of ~4.1%. Of note, aMAP score was missing for 575/1908 (30.1%) participants (see Table 1).

**TABLE 1:** Characteristics of study population.

Characteristic		*n* (col %)
baseline		
Age, mean (sd)		55.0 (sd: 9.2)
Gender	Female	506 (26.5)
	Male	1402 (73.5)
Ethnicity	White	1542 (80.8)
	non‐White	366 (19.2)
Decompensated cirrhosis, *n* (col%)	No	1529 (80.1)
	Yes	379 (19.9)
Follow‐up at transplant clinic	No	1105 (57.9)
	Yes	803 (42.1)
Alcohol hospital admission, *n* (col%)	No	1450 (76.0)
	Past	227 (11.9)
	Recent	231 (12.1)
Substance misuse hospital admission, *n* (col%)	No	1102 (57.8)
	Past	348 (18.2)
	Recent	458 (24.0)
IFN free therapy	No	472 (24.7)
	Yes	1436 (75.2)
Year SVR achievement, median (IQR)		2015 (IQR: 2014–2016)
aMAP score at SVR, median (IQR)		61.3 (IQR: 55.9–65.9)
***Follow‐up***		
Median duration of follow‐up (years)		3.8 (IQR: 3.3–4.9)
Total number of ultrasound scans		10 376
Number of incidents HCCs		133

*Note*: amap indicates risk of HCC at SVR achievement. A value of 61.3 suggests 3‐year HCC probability of 4.1%. However, amap score at this time point was missing for 575 (30.1%) of participants.

The median duration of follow‐up was 3.8 years per patient, ranging from 1 to 8 years. Most patients achieved SVR between 2014 and 2016.

### 3.3 Uptake of biannual US

**FIGURE 2:**
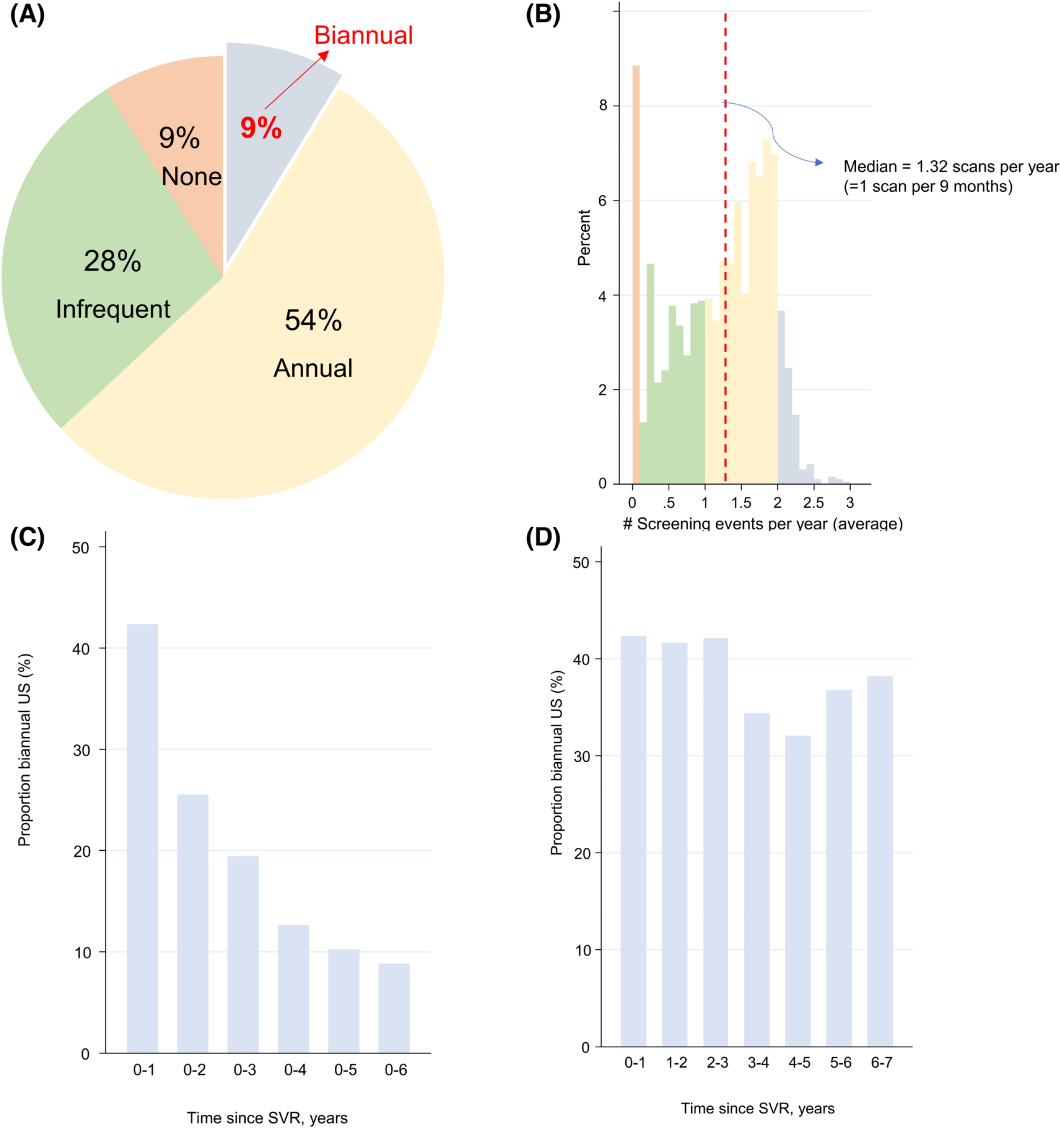
Summary of US uptake: (A) uptake category; (B) distribution of uptake rate; (C)uptake by duration Follow‐up; (D) uptake within 1‐year time bands.

In total, there were 10 376 scans observed during follow‐up translating into 9309 screening episodes (Figure 1). The proportion who received biannual US was 8.8% (*n* = 168). Otherwise, 54% (*n* = 1035) received annual surveillance; 28% (*n* = 536) received infrequent surveillance; and 9% (*n* = 169) received no surveillance (Figure 2).

The proportion with biannual surveillance increased to 19.7% and 29.0% when defined as ≥1 scan per 6.5 months and ≥1 scan per 7 months respectively. It was also sensitive to the window period used to define contiguous scans (i.e., 15.7% with a shorter 30‐day interval versus 4.0% for a longer 150‐day window) and increased too if CT and MRI scans were included to 12.4% (Figure S2).

Biannual uptake was highest over shorter time periods (i.e., 42% in the first year following SVR versus 19% in the first 3 years after SVR). This is because it is easier to be adherent to biannual US over a shorter time period than a longer one. However, within 1 year time bands, the proportion with biannual US was relatively constant with time, albeit highest in the first 3 years after SVR (Figure 2).

### 3.4 Factors associated with biannual US

Three main factors were associated with biannual US in multivariate regression analysis (Table 2). First, older patients were more likely to receive biannual US than younger patients (aOR per 10‐year increase: 1.41; 95% CI: 1.17–1.71; *p* < .001). Second, patients with decompensated cirrhosis had greater odds versus patients with compensated cirrhosis (aOR: 1.64; 95% CI: 1.10–2.43; *p* = .02). Third attendance at a liver transplant centre was associated with greater uptake (aOR: 3.41; 95% CI: 2.40–4.83; *p* < .001).

**TABLE 2:** Factors associated with biannual screening uptake.

Characteristic		Uptake, %	Univariate analysis		Multivariate analysis	
			OR	*p*	aOR	*p*
Age, per 10‐year increase		NA	1.57 (1.31–1.87)	<.001	1.41 (1.17–1.71)	<.001
Gender	Female	9.3	REF (1.00)	–	REF (1.00)	–
	Male	8.6	0.92 (0.65–1.31)	.65	1.08 (0.74–1.57)	.70
Ethnicity	White	8.4	REF (1.00)	–	REF (1.00)	–
	Non‐White	10.4	1.26 (0.86–1.84)	.24	1.09 (0.73–1.64)	.67
Decompensated cirrhosis	No	7.9	REF (1.00)	–	REF (1.00)	–
	Yes	12.4	1.65 (1.15–2.36)	.006	1.64 (1.10–2.43)	.02
Alcohol hospital admission	No	9.7	REF (1.00)	–	REF (1.00)	–
	Past	4.8	0.48 (0.25–0.90)	.02	0.51 (0.26–1.00)	.05
	Recent	7.4	0.74 (0.44–1.25)	.27	0.81 (0.44–1.50)	.51
Substance misuse hospital admission	No	10.1	REF (1.00)	–	REF (1.00)	–
	Past	8.0	0.78 (0.51–1.20)	.26	1.05 (0.66–1.67)	.84
	Recent	6.3	0.60 (0.40–0.92)	.02	0.69 (0.42–1.12)	.13
Follow‐up at transplant clinic	No	4.7	REF (1.00)	–	REF (1.00)	–
	Yes	14.4	3.42 (2.43–4.81)	<.001	3.41 (2.40–4.83)	<.001
Follow‐up duration, per 1‐year increase		NA	0.73 (0.64–0.82)	<.001	0.72 (0.63–0.82)	<.001

*Note*: Statistically significant associations are highlighted in grey.

Individuals with a past alcohol‐related hospital admission were less likely to receive biannual US versus those without (aOR: 0.51; 95% CI: 0.26–1.00; *p* = .05). There was also a trend towards reduced uptake in individuals with a recent hospital admission for substance abuse, albeit this did not reach statistical significance (aOR: 0.69; 95% CI: 0.42–1.12; *p* = .13). Gender and ethnicity were not associated with biannual surveillance. All associations remained broadly similar in sensitivity analyses (Table S2).

In a post hoc analysis, the association between attending a transplant centre and receiving biannual US did not attenuate after adjusting for HCC risk (i.e., via aMAP score) (Table S3). This analysis also suggested individuals with a higher risk of HCC were more likely to receive biannual US (aOR per 1 unit increase in aMAP: 1.06; 95% CI: 1.03–1.09; *p* < .001).

### 3.5 Variability in biannual US between liver centres

**FIGURE 3:**
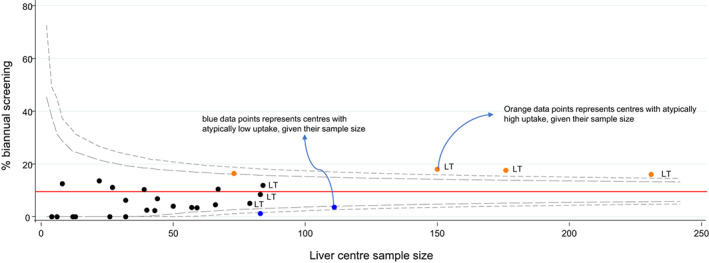
Funnel plots indicating the proportion of patients receiving biannual surveillance by liver centre. Liver centres are represented by circular data points. Liver transplant centres are marked “LT”. The red horizontal line is the average uptake for all the data points represented in the plot. The grey dashed line refers to the 95% and 99% control limits, calculated using the exact method. Data points for two centres (centre “S” and “K”) were omitted from this plot. Please see appendix A for further details.

The crude proportion of patients who received biannual uptake varied from 0% to 18% by liver centre. Funnel plots indicated statistically significant heterogeneity in biannual uptake between individual centres (Figure 3). Two centres with poor validation data were omitted from this plot (see Appendix A for further details). Of the 29 centres remaining, 6 (21%) were outside the 95% control limits (i.e., 4 centres above and 2 centres below). Inter‐centre variability was even more pronounced in the first 3 years after SVR, where 10/29 (34%) centres were outside control limits (Figure S3).

### 3.6 Relationship between biannual screening and curative HCC treatment

**FIGURE 4:**
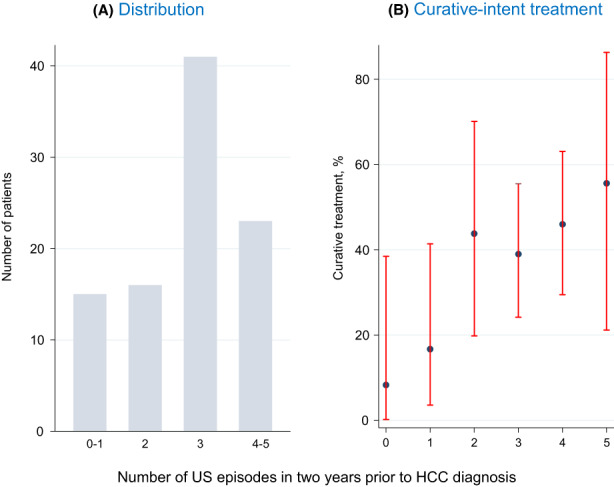
Ultrasound episodes two years prior to HCC diagnosis (*N* = 133).

133 incident cases of HCC were observed during follow‐up. The HCC incidence rate was 2.2 events per 100 person years (95% CI: 1.8–2.6). Of the 133 incident HCC cases observed, 37% (*n* = 49) were treated for HCC with curative intent. The proportion treated with curative intent increased roughly stepwise with number of US episodes received in the two years prior to HCC diagnosis; that is 8%, 17%, 44% 39% 46% and 56% in patients with 0, 1, 2, 3, 4 and 5 US episodes respectively (*p* = .06) (Figure 4).

**FIGURE 5:**
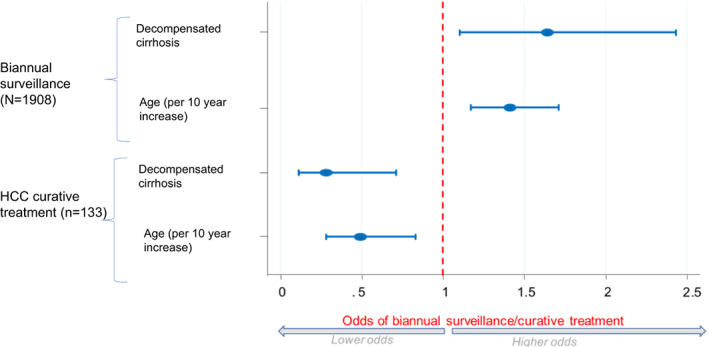
Older age and decompensated cirrhosis are associated with higher odds of biannual surveillance, but lower odds of HCC curative treatment.

In multivariate analysis, the odds of curative intent treatment increased by 53% for each additional US episode performed (aOR: 1.53; 95% CI: 1.12–2.09; *p* = .007). Conversely, older age (aOR per 10‐year increase: 0.49; 95% CI: 0.28–0.83; *p* = .008) and decompensated cirrhosis at SVR (aOR: 0.28; 95% CI: 0.11–0.71; *p* = .007) were associated with lower odds of curative treatment (Table 3; Figure 5).

**TABLE 3:** Factors associated with receiving curative HCC therapies (*N* = 133).

Characteristic		Univariate analysis		Multivariate analysis	
		OR	*p*	aOR	*p*
# USs in previous 2 years (odds for each additional US performed)		1.52 (1.14–2.03)	.004	1.53 (1.12–2.09)	.007
Age, per 10‐year increase		0.62 (0.38–0.99)	.047	0.49 (0.28–0.83)	.008
Gender	Female	REF (1.00)	–	REF (1.00)	–
	Male	1.82 (0.74–4.47)	.19	1.40 (0.53–3.72)	.50
Decompensated cirrhosis	No	REF (1.00)	–	REF (1.00)	–
	Yes	0.32 (0.14–0.73)	.007	0.28 (0.11–0.71)	.007

*Note*: Statistically significant associations are highlighted in grey.

In a post hoc subgroup analysis, higher aMAP score at SVR was associated with lower odds of curative‐intent treatment, albeit the association did not reach statistical significance (aOR per 1 unit increase in aMAP: 0.93; 95% CI: 0.986–1.01; *p* = .086).

### 3.7 Validation

The median time interval between successive screening events was 182–189 days (Figure S4). The date of HCC diagnosis coincided with a peak in the number of imaging procedures performed (Figure S5). Overall, we show NHS digital had adequate validity for measuring US uptake in cirrhosis patients. Detailed information can be found in Appendix A.

## 4 DISCUSSION

This study describes the delivery of biannual US screening in a large multi‐centre cohort of patients with cured hepatitis C in the UK. Our analysis raises several important and novel points regarding the implementation of HCC surveillance in the UK. First, the identification of >10 000 US scans suggests appreciable resources are in fact being deployed towards early HCC detection in this population. Nevertheless, few patients are receiving biannual US as recommended in guidelines^9^ – that is only 19% in the first three year after SVR achievement and 9% during all years of follow‐up. Second, the odds of biannual screening were ~ 3 times greater for patients attending a liver transplant centre. This suggests that rather than being delivered equally to all eligible patients, US uptake is influenced by arbitrary factors such as the type of liver clinic one is attending. In a similar vein, funnel plot analyses showed significant variability in uptake of biannual US between individual liver centres. To the best of our knowledge, this is a novel finding that raises important questions regarding equity of access. Third, our study describes the HCC care cascade in unique detail, from surveillance of at‐risk patients through to receipt of curative‐intent treatment. Our findings highlight the low efficiency of biannual US. For example, 10 376 US scans were performed in this cohort, to ultimately treat only 49 patients with curative intent. It should be pointed out that not all US scans are performed exclusively for HCC surveillance (e.g., US is also used to detect mild ascites in patients with cirrhosis^18^); nevertheless, even with this in mind, the yield is still very low. Our data suggest biannual US is not currently being targeted in the most appropriate way. Indeed, we show there is a disparity between individuals who are currently being prioritised for biannual US versus those who are good candidates for curative‐intent treatment. For example, on the one hand, older patients were more likely to receive biannual US, but on the other hand, they were less likely to be treated with curative intent if they did develop HCC (Figure 5). The same pattern applied to decompensated disease and may extrapolate to HCC risk in general. These observations caution that focusing screening on higher risk patients may not necessarily translate into more patients being treated for HCC with curative intent. This has implications for the current debate around individualised HCC surveillance.^19^, ^20^, ^21^, ^22^ Fourth, our study suggests the more US scans you receive in the two years prior to HCC, the greater your odds of receiving curative‐intent treatment are. This supports the fundamental premise of biannual US and reinforces the potential benefits for patients. However, further work is needed to articulate the net benefit of surveillance to patients and clinicians in terms of years‐of‐life‐gained, and how this may vary for different patient groups (e.g., older patients). This information is crucial to support shared decision making.^23^ Without a randomised controlled trial, this may be best established using decision modelling methods.^24^, ^25^ Finally, we demonstrate the validity of the NHS England DID for quantifying uptake of biannual US in patients with cirrhosis. Future studies may consider linking the DID to broader datasets such as the clinical practice research datalink.^26^ In this way, one could repeat this study for a much larger set of patients with a range of cirrhosis beyond just hepatitis C.

Our study has several limitations that merit discussion. First, we did not have any information on the medical indication for the imaging procedures included. Many of the scans considered may not meet a strict definition of HCC surveillance. For example, they may have been performed in response to symptoms (e.g., weight loss or changes in liver blood test values), or with more than one medical indication in mind (e.g., detecting mild ascites in addition to checking for focal lesions on the liver^18^). Nevertheless, the aim of this study was to assess adherence to clinical guidelines, which simply recommend biannual US checks of the liver/abdomen for focal HCC lesions.^7^, ^8^, ^9^ In this sense, it does not matter if the US was prompted by symptoms or if it was carried out with an additional objective in mind – as long as the US provided an opportunity to detect HCC. Second, we did have any data on BCLC stage at HCC diagnosis and thus had to rely on curative treatment as a marker of early HCC detection. Third, a patient's liver centre was defined as the liver centre overseeing their care at the time of enrolment into HCVRUK. However, this may not necessarily be the same centre overseeing care at the time of SVR achievement. Thus, there may be some misclassification with respect to this variable. In addition, we did not exclude patients with advanced liver cirrhosis (i.e., Child‐Pugh C) that were not on the waiting list for liver transplantation at the time of SVR achievement. Such patients would not be eligible for HCC screening as per EASL guidelines.^8^ However, they represent a relatively small patient subgroup and so their inclusion is unlikely to have biased our results. Another caveat to note is that most patients in this study were treated for HCV in specialist care settings. More recently however, there has been a shift towards treating patients in the community where it is even more difficult to engage patients in HCC surveillance. In this respect, the low surveillance uptake observed in this study may even be on the optimistic side. Another limitation is that some participants in this cohort may have emigrated – in which case, the imaging and outcome data will not be reliable. Further, we did not have information on social factors – such as deprivation, household income or education – which may influence uptake of biannual US. Finally, we did not have recourse to detailed information about individual liver centres (e.g., staffing, number of dedicated hepatologists), to permit a more thorough investigation into inter‐centre variability.

Overall, this study provides important insight into how HCC surveillance is being delivered in the UK. Our findings argue for greater standardisation in delivery and arguably a need to monitor HCC surveillance routinely at a national level.

## FUNDING INFORMATION

HCVRUK was established through a grant from Medical Research Foundation (Grant ID: C0365). The Medical Research Foundation also supported this study via a viral hepatitis fellowship awarded to HI (Grant ID: C0825). The STOP‐HCV study was funded by the Medical Research Council, United Kingdom (Grant ID: MR/K01532X/1). This work has also been supported by the Deliver study, funded by Cancer Research UK (Grant ID: C30358/A29725). The funders had no involvement in the study design; in the collection, analysis and interpretation of data; in the writing of the report; and in the decision to submit the article for publication.

## CONFLICT OF INTEREST STATEMENT

There are no relevant conflicts of interest to report.

## Supporting information


Data S1
