# Supplementary material for: Delivery of biannual ultrasound surveillance for individuals with cirrhosis and cured hepatitis C in the UK
Source: Liver Int. 2023 Feb 20;43(4):917–27. doi: 10.1111/liv.15528 (PMC10946603; doi:10.1111/liv.15528)
Supplement: Supplementary file 1 — Data S1 [file LIV-43-917-s001.docx]

**Delivery of biannual ultrasound surveillance for individuals with cirrhosis and cured hepatitis C in the UK**

AUTHORS: Victoria Hamill; Will Gelson; Douglas MacDonald; Paul Richardson; Stephen D. Ryder; Mark Aldersley; Stuart McPherson; Sumita Verma; Rohini Sharma; Sharon Hutchinson; Jennifer Benselin; Eleanor Barnes; Indra Neil Guha; William L. Irving; Hamish Innes.

Table of contents

Supplementary Figure 1………………………………………………………….…..2

Supplementary Figure 2………………………………………………………….…..3

Supplementary Figure 3………………………………………………………….…..4

Supplementary Figure 4………………………………………………………….…..5

Supplementary Figure 5………………………………………………………….…..6

Supplementary Figure 6………………………………………………………….…..7

Supplementary Figure 7………………………………………………………….…..8

Supplementary Figure 8………………………………………………………….…..9

Supplementary Table 1………………………………………………………….10-11

Supplementary Table 2……………………………………………………………..12

Supplementary Table 3……………………………………………………………..13

Supplementary Table 4……………………………………………………………..14

Appendix A………………………………………………………………………..15-17

**
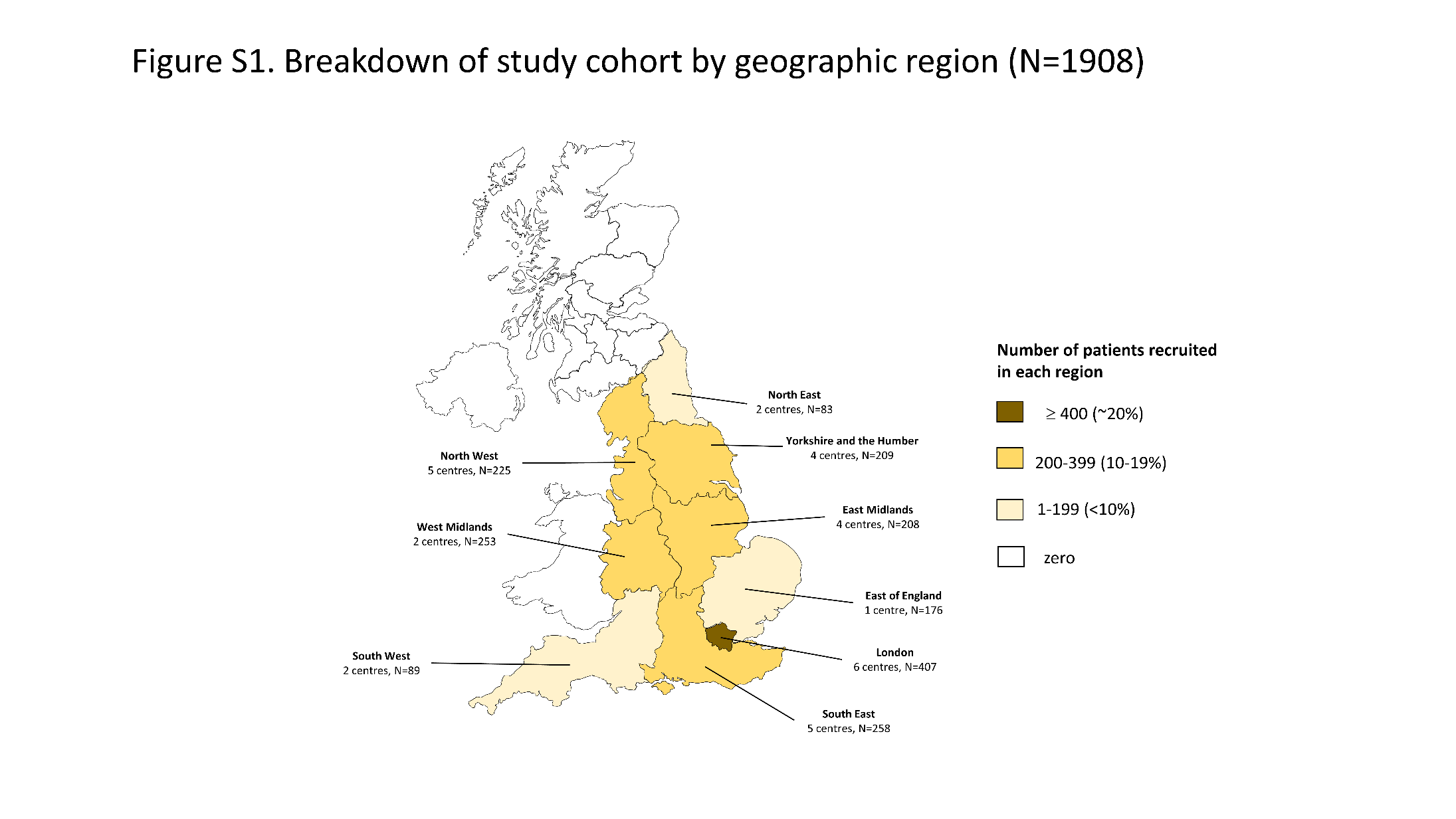
**

**
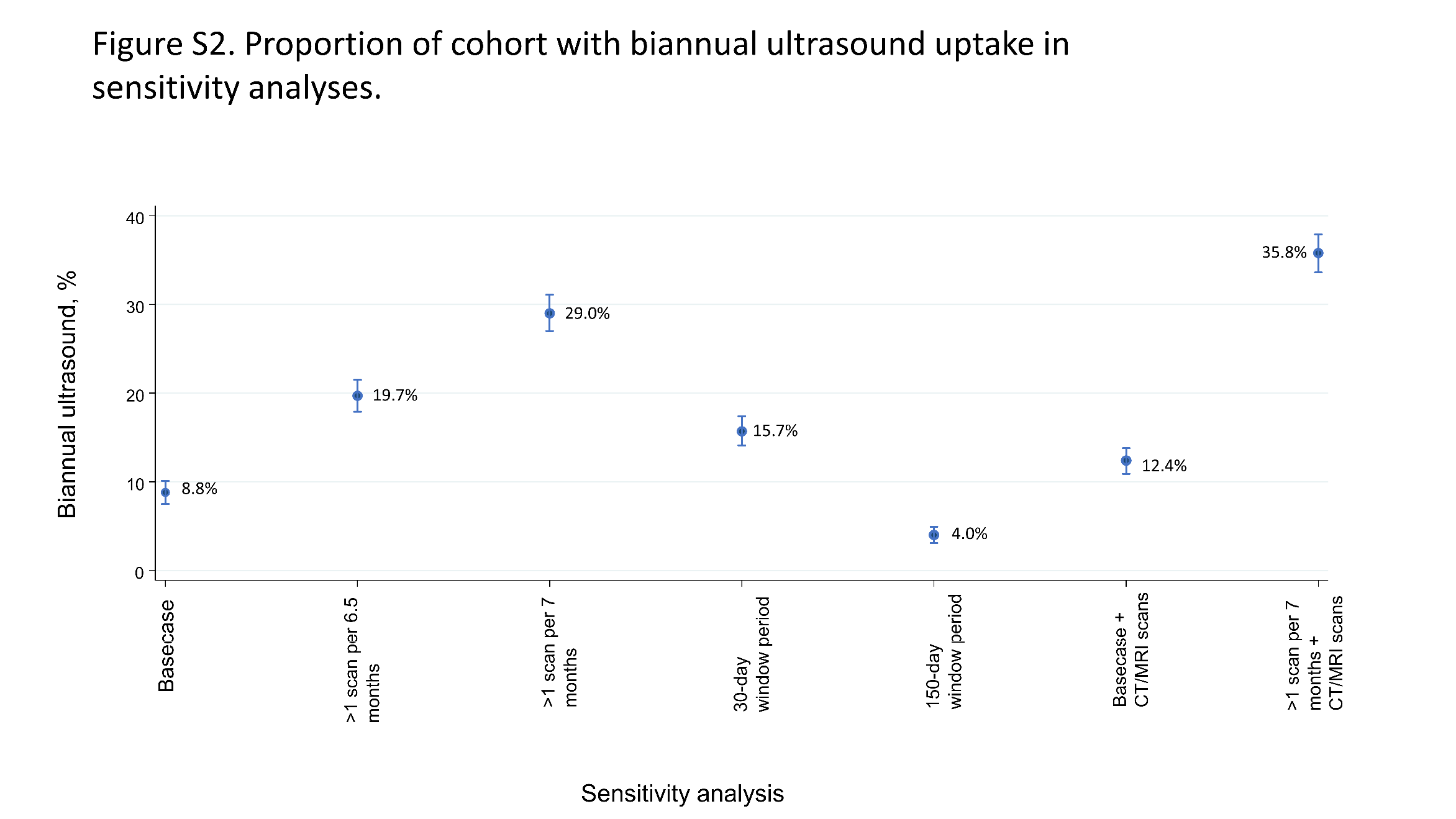
**

**
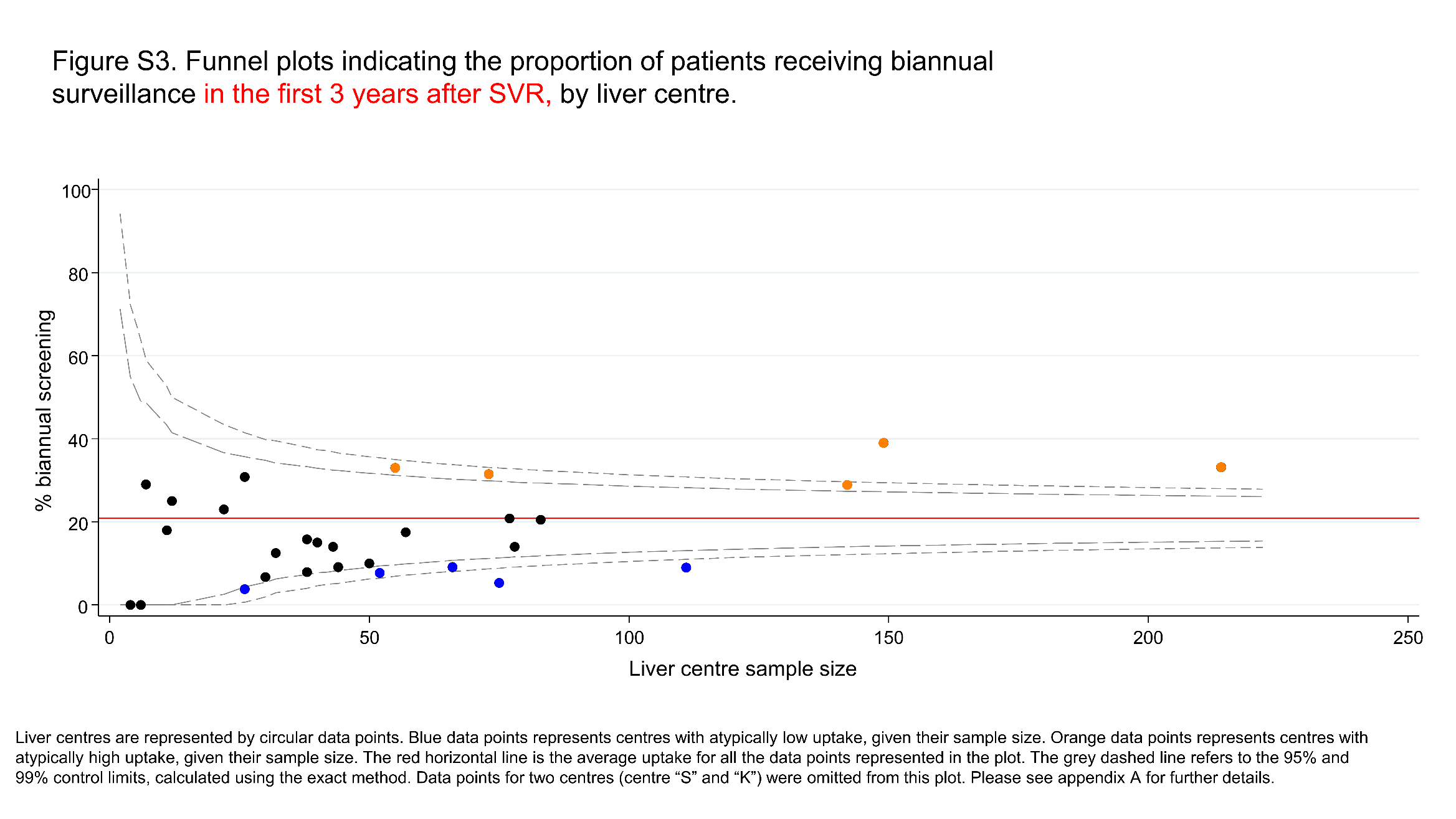
**

**
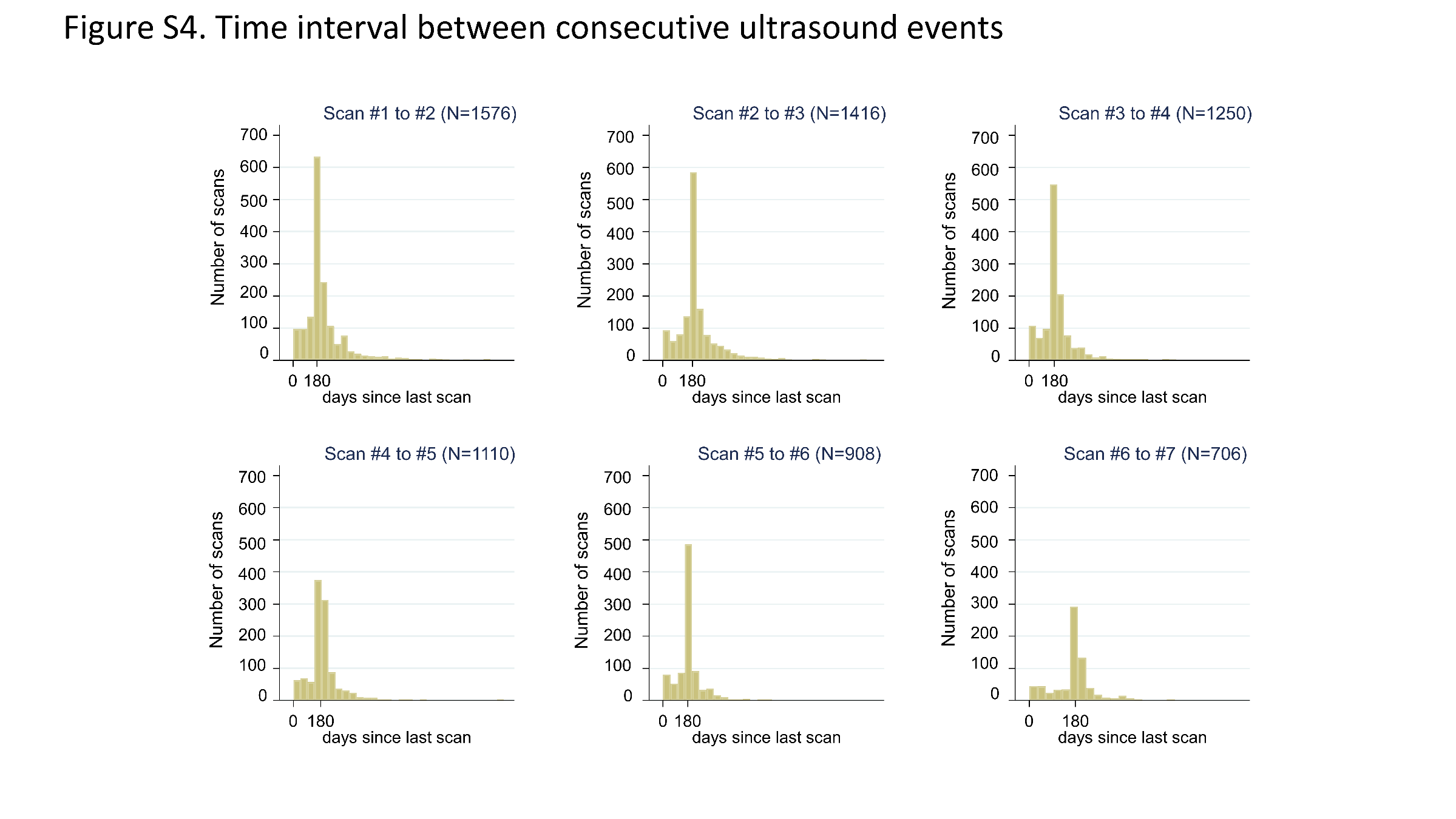
**

**
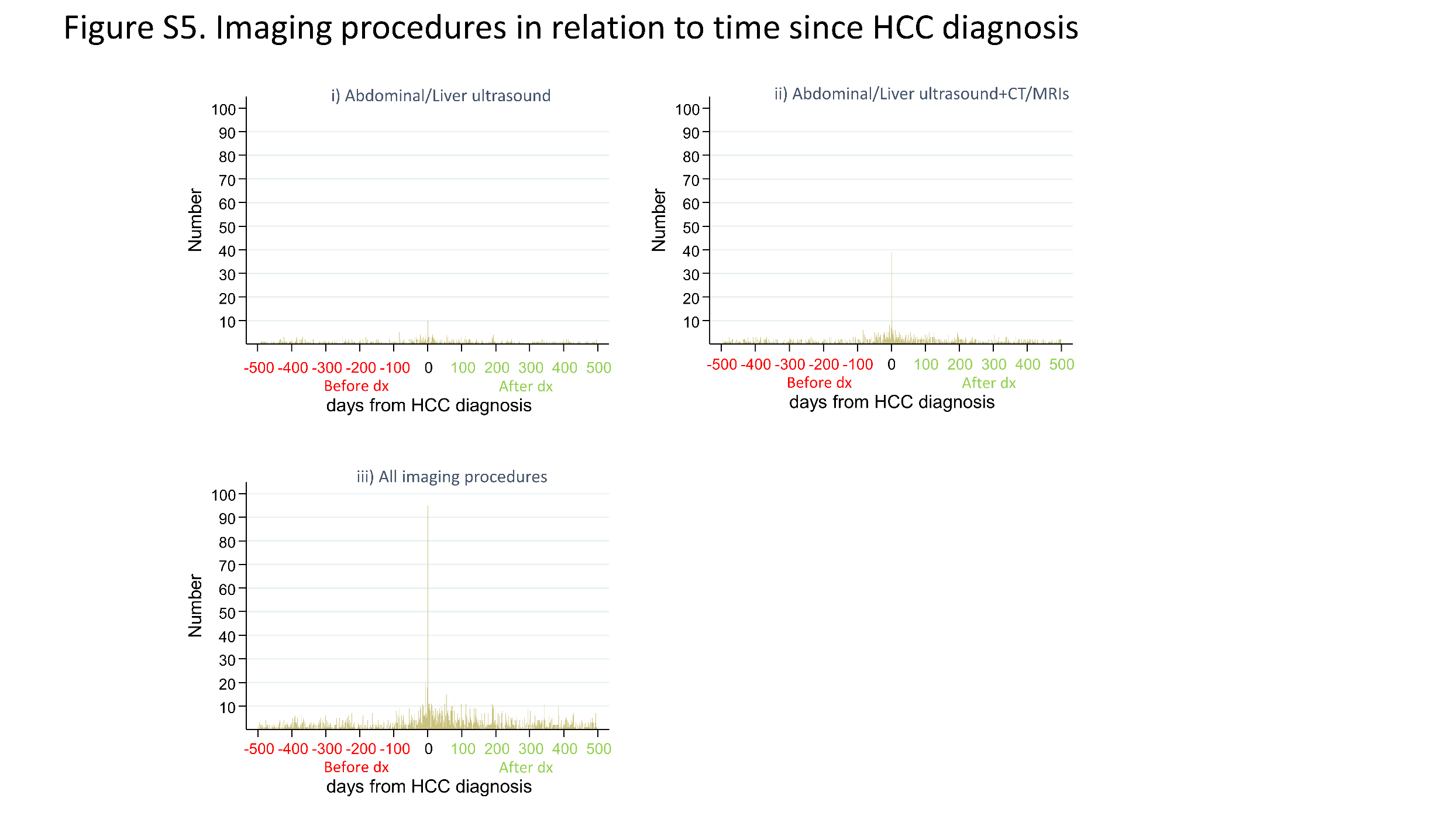
**

**
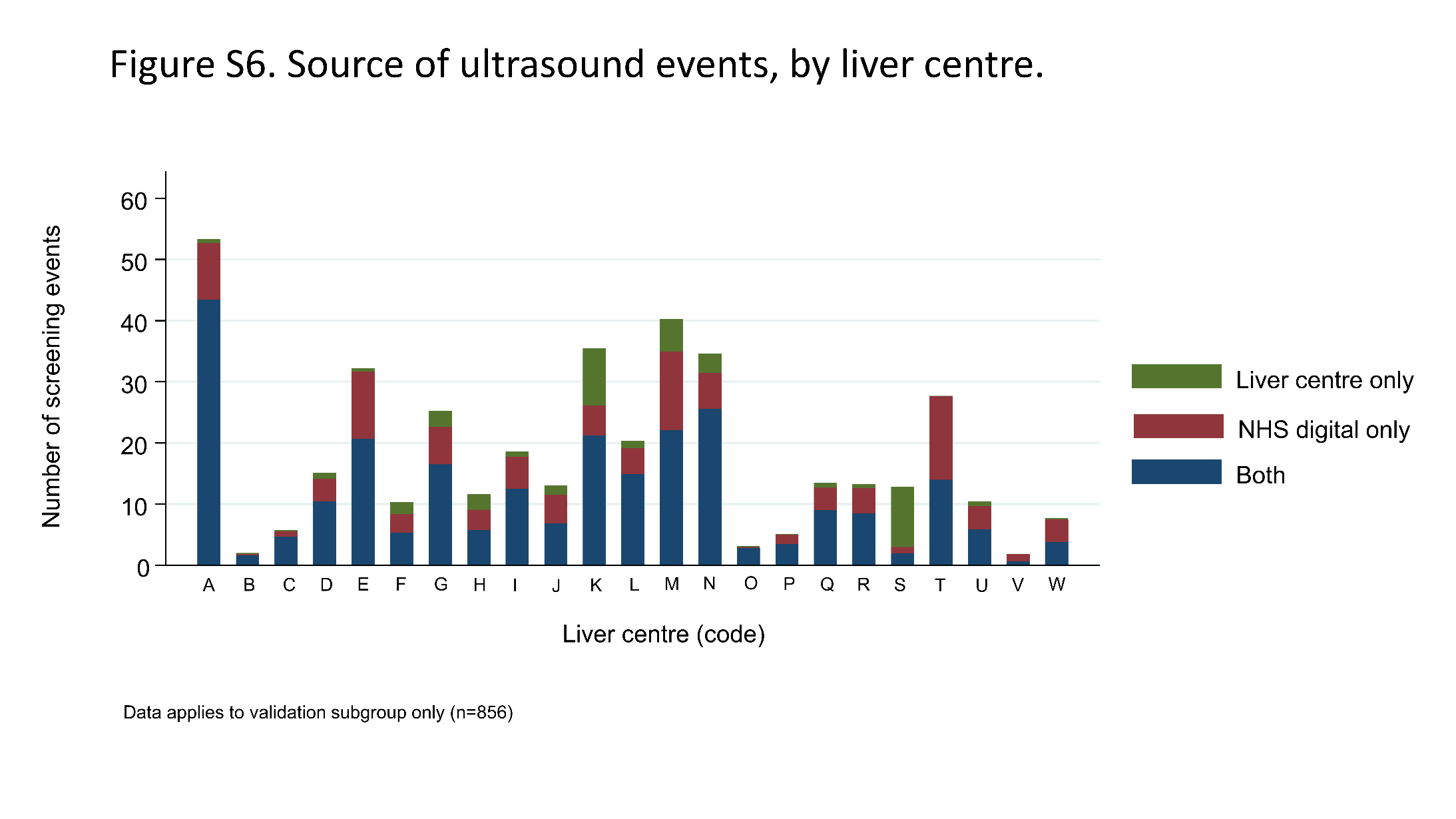
**

**
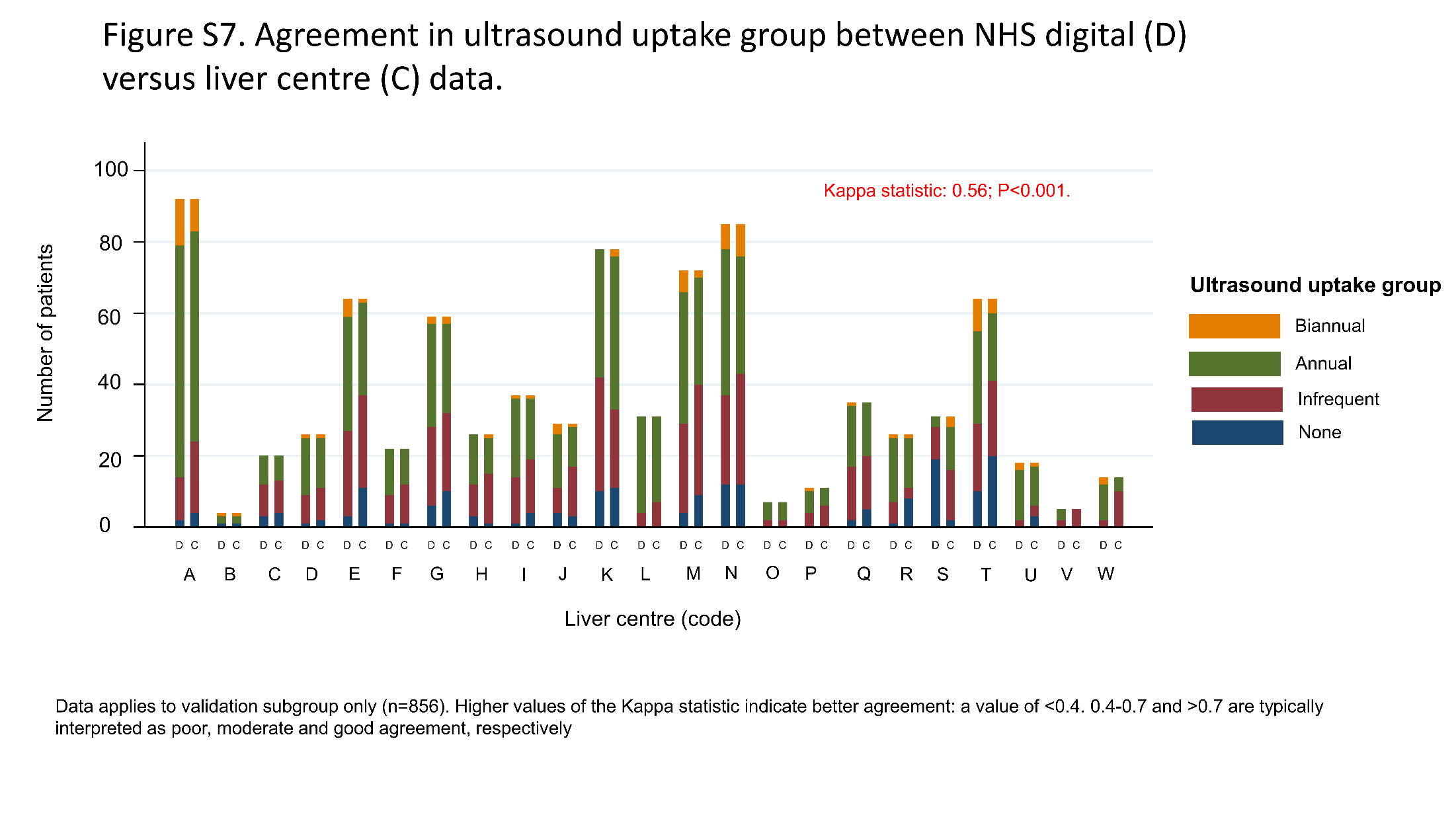
**

**
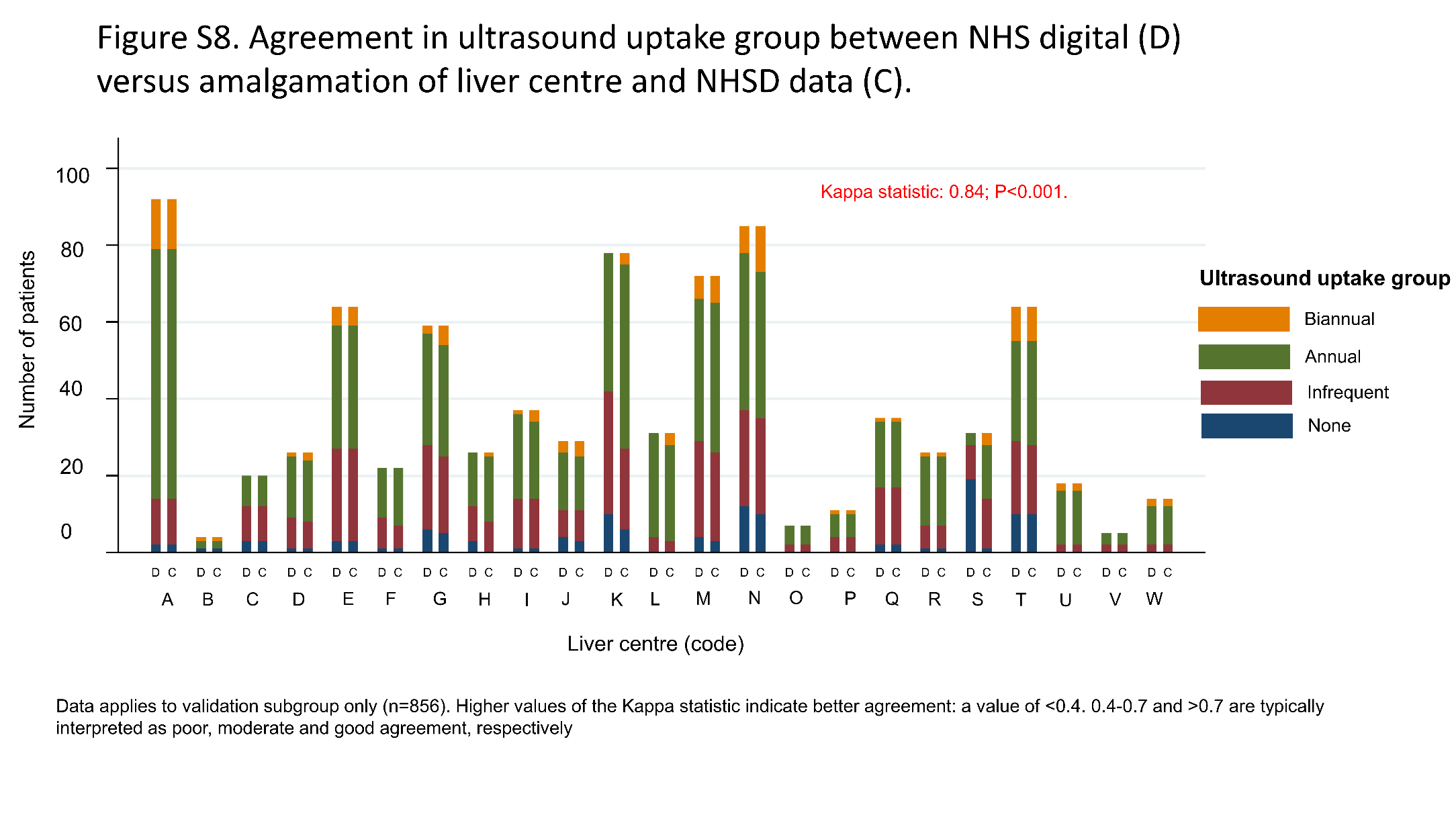
**

**APPENDIX A: VALIDATION OF NHS DIGITAL DATA**

**1.0 Methods:**

Internal and external validation approaches was performed to assess the validity of NHS digital data to measure uptake of abdominal/liver ultrasound (US) events in cirrhosis patients.

**1.1 Internal validity**

First, the average time interval between consecutive US scans was calculated to assess consistency with the screening interval recommended in clinical guidelines.

Second, we assessed the timing of imaging examinations in patients who developed HCC.

**1.2 Agreement with data collected from individual centres**

Moreover, for a subset of patients, we collected information on liver/abdominal US scans directly from liver centres. On behalf of the HCVRUK, we contacted centres directly to request the dates of US examinations performed after SVR achievement for patients in this study population. Centres then retrieved these dates manually by interrogating local patient information systems by clinicians and/or data entry personnel. Not all centres responded to this request however, and thus data were only available for a subset of the total study population. Dates of US scans from NHS digital were then amalgamated with those collected from individual liver centres. Scans occurring on the same date were regarded as duplicates. Contiguous scans occurring within 90 days of one another were collapsed into a single screening episode, as described previously. We then assessed agreement between US dates supplied by individual liver centres versus those ascertained through NHS digital data. We did this both visually (bar charts) and quantitatively (Kappa statistic). In general, higher values of kappa indicate better agreement; a value of <0.4, 0.4-0.7 and >0.7 are typically interpreted as poor, moderate and good agreement, respectively.

We also assessed the agreement between biannual US uptake inferred from NHS digital data versus biannual uptake inferred by amalgamating dates from NHS digital + individual centres.

**2.0 RESULTS**

**2.1 Internal validity**

The median time interval between successive US scans (i.e. US events) was 182-189 days. (Figure S4).

Imaging procedures were clustered around the date of HCC diagnosis (Figure S5). This was most pronounced for CT/MRI scans and for all imaging procedures. The most common non-liver imaging procedure within 10 days of HCC diagnosis was a plain chest X-ray. This implies frequent HCC detection following pulmonary metastasis, consistent with Katyal S, et al. Radiology. 2000;216:698-703.

**2.2 Agreement with data collected from individual centres**

**2.2.1 Combining scans provided by liver centres with scans from NHS digital**

We collected dates of US events directly from individual centres for a subgroup of 856/1908 (44.8%) patients. The characteristics of this subgroup are shown in Table S4. In total, 4131 US events were identified after pooling the data collected from individual centres with data from NHS digital. The provenance of these events were: 433 (10%) reported by liver centres alone; 1079 (26%) reported by NHS digital data only; and 2619 (63%) reported by both the liver centre and the NHS digital data. However, these proportions did vary by clinic. In clinic “A” for instance, only 6/533 (1%) events were sourced from the clinic alone, whereas for clinic “S”, 98/128 (77%) events were reported by the clinic alone. Clinic “K” also exhibited a high proportion of events sourced from clinic data alone (Figure S6).

**2.2.2 Quantifying agreement:**

The proportion with biannual US uptake was 4.6%, 6.4% and 9.1% when inferred from individual centre data only, NHS digital data only, and an amalgamation of the two, respectively.

With respect to biannual US uptake, the overall agreement between NHS digital data Vs individual centre data was moderate (kappa statistic 0.56; P<0.001) (Figure S7).

With respect to biannual US uptake, the overall agreement between NHS digital data Vs NHS digital + individual centre data was good (Kappa statistic: 0.84; P<0.001). (Figure S8)

**3.0 Interpretation**

Overall, NHS digital data showed good validity for measuring US uptake in patients with cirrhosis. However, some blind spots for patients attending specific liver centres. In clinic “K” for example, biannual uptake was 0% based on NHS digital alone versus 4% when based on NHS digital + individual centre data. Subsequent local investigation of clinic “K” showed that the ultrasound scans missed by NHS digital were all performed by one specific non-NHS provider. The most likely explanation therefore is that this specific provider was not contributing data to the DID, even though the services carried out were commissioned by the NHS. The data for clinic “S” also appeared to be unreliable. Despite these gaps in the NHS digital data, the additional data from liver centres did not change the broad picture on screening uptake. For example, in the validation cohort, the proportion of patients with biannual uptake increased only modestly after adding centre data into the mix (i.e. from 6.4% when based on NHS digital data only, to 9.1% when based on NHS digital data + data from individual centres). Moreover, the agreement between biannual US uptake when inferred from NHS digital Vs. NHS digital + centre data was very strong with a Kappa statistic >0.80. This justifies our reliance on the NHS digital alone in our main analysis.
